# The assessment of mortality and quality of life after intertrochanteric fracture of femur in patients older than 60 at Emam Khomeini Hospital of Ahvaz

**DOI:** 10.12669/pjms.334.13146

**Published:** 2017

**Authors:** Seyed Abdolhossein Mehdi Nasab, Ebrahim Khorramdin

**Affiliations:** 1 Dr. Seyed Abdolhossein Mendi Nasab, MD. Professor of Orthopaedic Surgery, Department of Orthopedic Surgery, Emam Khomeini Hospital, Azadegan Street, Ahvaz Jundishapur University of Medical Sciences, Ahvaz, Iran; 2 Dr. Ebrahim Khorramdin, MD. Orthopaedic Surgeon, Department of Orthopedic Surgery, Emam Khomeini Hospital, Azadegan Street, Ahvaz Jundishapur University of Medical Sciences, Ahvaz, Iran

**Keywords:** Intertrochanteric fracture, Life quality, Morbidity

## Abstract

**Objective:**

To investigate the mortality and disability rates after surgical treatment of intertrochanteric fractures in patients older than 60 years old.

**Method:**

In this retrospective study, 385 patients aged 60 or older who were admitted because of intertrochanteric fracture to treatment and teaching center of Emam Khomeini hospital of Ahvaz, Iran between Mar. 2010 to Feb. 2015 and underwent surgery were included. All the patients were treated by open reduction and internal fixation by dynamic hip screw. Two hundred and six patients were men (53.5%) and 179 were women (46.4%). Age of patients was between 60 to 89 years old with the average of 75.2 years old. Minimum time required after surgery to enter this study was one year. Results were gathered based on examination of patient or calling patients and their families by phone number.

**Result:**

Rate of mortality was 36.9%, including 54.9% for men and 41.9% for women. In eleven patients (2.85%), initial reduction was lost because of failure of fixation device. For these patients reoperation was performed, and 7 of them (63.63%) expired within the mean of 10.1 months after reoperation. Time delay for surgery after occurrence of the fracture was in range of 2 to 15 days with an average of 4.8 days.24 patients (6.23%) went under surgery later than one week after fracture had been happened which seven of them (29.16%) expired. Highest mortality rate was in the age group of 80-89 years old with 50 patients (63.01%) and lowest one was in the group of 60-69 years old with three patients (4.67%). Disability rate and quality of life of the patients was measured by Modified Harris Hip Score and divided in 3 group of good (with a score of 71 to 90), fair (with a score of 51 to 70) and weak (with a score of 0 to 50). Patients who had good score consisted of 35.54% of patients with the average age of 64.63 years old and majority of male patients, fair group consisted of 30.5% of patients with average age of 73.45 years old and equal percentage of male and female patients and for Weak group it was 34.2% and 73.45 years old and by majority of male patients.

**Conclusion:**

Mortality rate of intertrochanteric fracture of femur is high even after treated with surgery and it’s highly related to age of patient. Furthermore, quality of life after surgery is still low and follow up of the patients should be improved.

## INTRODUCTION

Intertrochanteric fracture represents a major public health problem and is the most serious complication of osteoporosis in elderly persons. The incidence of this fragility fracture greatly varies among different countries and rates in studies have been reported from 37 to 399.3 per 100000 population / year for men and 97.5 to 920.7 per 100000 / year for women.^1^

This sort of fracture accounts for 7% of entire osteoporotic fractures.^2^ The intertrochanteric hip fractures generally cross in the area between the greater trochanter (the junction of abductor and extensor) and lesser trochanter (the junction of flexor muscles).^3^ These types of fracture are prevalent in older people with osteoporosis following minor trauma and contributes to both morbidity and mortality in the elderly patients. In younger patients, these fractures are usually associated with high-energy and multi-trauma injuries. About 90% percent of intertrochanteric fractures can be seen in people over 65 years old.^4^,^5^ This event in the elderly population is associated with high rates of mortality and a drastic decrease in performance^6^ and a drop in the quality of life.^7^

Individuals suffering from intertrochanteric fractures should be mobilized as soon as possible; otherwise, they would be at a risk of serious complications such as bedsores, urinary tract infections, joint stiffness, pneumonia and thromboembolism. The major therapeutic strategy in this regard is the fracture reduction and internal fixation with Dynamic Hip Screw (DHS) but some fixation devices such as angled blade plates, Ender’s nail or cephalomedullary nail can be recruited in certain circumstances. Non-surgical treatments of intertrochanteric fracture, such as traction or early ambulation of patients, without fracture fixation are rarely used in some occasions that the patient is unable to walk previously or when the possibility of mortality is high.^8^ The DHS is a recommended treatment to fix the intertrochanteric fractures.^9^

One of the main problems to manage this type of fracture is the low level of patient’s return to the daily activities and ability to perform regular tasks before surgery.^10^ Fifty percent of patients need help to fulfill their daily tasks and about 25% should be cared long-term.^11^

There is high prevalence of osteoporosis in modern societies and the high probability of the occurrence of intertrochanteric fractures in the elderly individuals, as well as the heavy economic, social, physical and mental costs of these fractures imposing to the patients. On the other hand, there is failure to return to previous levels of daily functioning in half of patients. The present study aimed to assess the treatment success rate in patient’s performance and to draw more attention to preventative methods.

## METHODS

This retrospective study was conducted on older patients over 60 years suffering from intertrochanteric fracture who underwent surgery and fixation by DHS device at treatment and teaching center of Emam Khomeini hospital of Ahvaz, Iran, during a period of five years from March 2010 to February 2015. Assessment of these patients was based on the medical records and interviews with patients or their first-degree relatives. The life quality of patients was measured by Harris Hip Score questionnaire. Exclusion criteria were as follows:

•Patients whose pathologic fracture was on the basis of primary tumor, metastasis or metabolic disorders.
•Patients suffered from multiple fractures and multiple trauma due to accidents.
•Incomplete information in the medical records, and dissatisfaction of patients and families to participate in the study and to respond questions. The study was approved by ethic committee at our university.

The study population consisted of 385 patients, including 206 (53.5%) male and 179 (46.5%) female, with an age range of 60 to 89 years and the mean age of 75.2±8.79 years. The mean age of deceased patients was 81.4 years.

## RESULTS

The results of the current study showed that 142 (36.9%) out of 385 studied patients had died, including 78 men (54.9%) and 64 women (45.1%). The patients were divided into three age groups of 60 to 69 years, 70 to 79 years and 80 to 89 years. The highest and the lowest mortality rates were in the Group of 80 to 89 years age (63.01%) and the Group 60 to 69 years (4.67%), respectively (Table-I).

**Table-I:** mortality rates of the patients.

*Age*	*Gender/Survival status*			

	*Male*		*Female*	

	*Alive*	*Died*	*Alive*	*Died*
60 to 69 years	57	3	45	2
70 to 79 years	41	25	46	20
80 to 89 years	30	50 (62.5%)	24	42 (63.63%)

Eleven patients had a reoperation due to surgical equipment failure, of which seven patients died. Twenty-four patients who had undergone surgery more than one week after the fractures, of which seven patients were deceased.

The life quality of the patients mentioned in three age groups was assessed three groups of good (71 to 90), fair (51 to 70) and poor (0 to 50) based on the questionnaire scoring. The Group good included 86 patients (35.54%) with a mean age of 64.63 years and most of them were men. The Group fair had 74 patients (30.5%) with a mean age of 73.45 years and with equal sex ratio. The Group poor involved 83 patients (34.20%) with a mean age of 77.45 years and most of them were men. In the age range of 60 to 69 years, 69.60% (n = 71) were in the Group good, 16.66% (n = 17) in the Group fair and 13.72% (n = 14) in the Group poor. The age range of 70 to 79 years consisted of 14.94% (n = 13) in the Group good, 49.42% (n = 43) in the Group fair and 35.63% (n = 31) in the Group poor. This rate in the age range of 80 to 89 years was 3.70% (n = 2) in Group good, 25.92% (n = 14) in Group fair and 70.37% (n = 38) in Group poor (Table-II).

**Table-II:** Quality of life of patients.

*Age range*	*Number of patients (percentage)*		

	*Good*	*Fair*	*Poor*
60 to 69 years	71 (69.6%)	17 (16.66%)	14 (13.72%)
70 to 79 years	13 (14.94%)	43 (49.42%)	31 (35.63%)
80 to 89 years	2 (3.7%)	14 (25.92%)	38 (70.37%)

The mean interval between the incidence of fracture and surgery was 4.8 days with a range of 2 to 15 days. Of 243 studied alive people, only four patients had reoperation (due to equipment failure) whose mean score (40.75) was lower compared to those with only once surgery (58.02) (Table-III).

**Table-III:** Device failure

*Questionnaire Score*	*No.*	*Mean Age*	*Gender*		*Fixation Device Failure*	*Surgery Duration (Week)*

			*Male*	*Female*		
Poor	83	77.45	43	40	2	6
Fair	74	73.45	37	37	1	8
Good	86	64.63	48	38	1	3

Moreover, 17 patients who were alive had surgery more than a week after fracture and the mean score of mHHS in these people was 55.52 and those who had undergone surgery less than a week had the mean score of 57.90.

## DISCUSSION

In this study, the majority of patients were male. However, the intertrochanteric hip fractures are reportedly more prevalent among females in most investigations; for example, 74.93% of patients were female in the study of Hindmarsh et al.^12^ This may be due to cultural differences so that older women are less interested in leaving the house, leading to less mobility.

In this study, 5-year postoperative mortality rate was 36.9% that is more than the study of Van Balen, which was 20%.^13^ However, the review duration in the mentioned study was only four months after fracture; high levels of mortality rate in the present study could be affected by time. Also in a study conducted by Scott Schnell, the mortality rate a year after fracture was reported 21.2%^14^, which this rate is clearly less than ours. In a study of Fierens, which was conducted between 2 groups in two 5-years periods from 1978 to 1983 and 1998 to 2003, the mortality rate was respectively 24% and 23%.^15^ This was far less than the current rate of 36.9%. This rate was reported 25.4% in the study of Pioli during a one-year period.^16^

In the study of Mellick J. Chehade et al. investigating the effects of stability of intertrochanteric fractures on mortality in the people aged 36-106 years, the respective rate was reported 30%. The reoperation rate at one year after surgery was 2.8%.^17^

This study similar to research of Kesmezacar et al. showed that the mortality risk is increased with aging and the mean age of deceased people was 81.5 years, which nearly is in line with the result of our study.^18^ The impact of mean interval between the incidence of fracture and surgery was also carried out the results revealed that delay in surgery probably increases the likelihood of complications.^17^ The reason for this delay could be related to the patient’s late referral to our hospital and also for treatment of their underlying diseases to ready for anesthesia and surgical procedure. However the delay due to surgery would not seem to have adverse effect on the mortality rates.

In the study of Hindmarsh^12^, which conducted on the mortality rate during 3 years among 16836 patients aged over 65 years, the mortality rate was 42.2% in men and 29% in women.^12^ In the group of over 85 years, this statics was 53.6% for men and 37.1% for women compared to similar statistics in people over 80 years, which was much smaller amounts in this study. This may be due to the higher quality of follow-up after surgery compared to our country.

In the current study, no significant relation was found between gender and mortality. However, in the studies of Hindmarsh and Kesmezacar, the mortality of male had higher rate.^12^,^18^ In the study of Pioli et al., the mortality in men was higher than in women. Probably, it was because of less attention that men pay to health before the fracture.

In the study of Van Balen, 43% of patients returned to previous ability to walk, while in the present study, 35.4% of subjects returned to good condition.^13^ This difference may be because of the weakness in health care and rehabilitation systems for the patients. Jorma et al in their study concluded that the risk of mortality in hip fracture patients was three fold higher than that in the general age group of population.^19^

In the study of Lee et al., 25% of the patients had great motion ability, and 40% of them were able to walk without any help.^20^ In addition, the results of Lee indicated the lack of remarkable impact of hip fracture on quality of life, but in this study, 34.2% of poor status of patients and 30.5% of fair status reflect the impact of these fractures on the life quality of the patients.

However, limitations and weaknesses of the study are needed to be addressed for obtaining more accurate results. For example, elderly patients were facing with the problems of difficult access and lack of follow-up, which were due to the difficulties in transportation of old patients and their dependence on relatives. In addition, we could not recognize the detailed cause of mortality and its relationship with the fractures. The effect and interference of underlying medical situations such as cardiovascular disease, diabetes, kidney failure, etc. on mortality rate were not determined.

## CONCLUSION

According the results obtained from 5-year follow-up of the patients, it can be concluded that mortality rate was higher than in other reports. In addition, quality of life for the patients had not improved as much as the other studies and it was lower than other studies. Regarding the respective high mortality rate of intertrochanteric fracture in this study, as well as its remarkable effect on quality of life, the best strategy appears to be the care, prevention of fractures and provision of rehabilitation services as well as correct follow-up of the patients in the next steps.
